# Crack-Templated Wire-Like Semitransparent Electrodes with Unique Irregular Patterns

**DOI:** 10.1021/acsomega.2c05131

**Published:** 2022-10-17

**Authors:** Anna M. Melnychenko, Robert Kudrawiec

**Affiliations:** † ŁUKASIEWICZ Research Network−PORT Polish Center for Technology Development, Stabłowicka 147, 54-066Wrocław, Poland; ‡ Department of Semiconductor Materials Engineering, Faculty of Fundamental Problems of Technology, Wrocław University of Science and Technology, Wybrzeże Wyspiańskiego 27, 50-370Wrocław, Poland

## Abstract

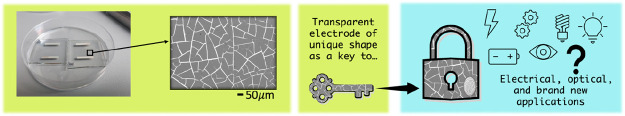

The development of novel methods of producing transparent electrodes is important because of their ever-evolving applications and thus the additional parameters they must meet. In this work, we present a method of manufacturing semitransparent silver electrodes. This technique involves cracking the polyvinylpyrrolidone layer in the presence of a colloidal nanodispersion of zinc oxide. The resulting cracked polymer layer serves as the disposable mask for metal deposition. The whole procedure is valuable due to the fast and easy step of cracks formation caused by the elevated temperature and reduced pressure. The obtained electrodes have high transparency (82.4%) in a wide spectral range, which is only limited by the transparency of the applied substrate, and low resistivity (27.3 × 10^–7^ Ωm). The presence of unique patterns suggests new ideas for the applications of such electrodes, such as coding, security, and antiplagiarism protection.

## Introduction

Transparent electrodes (TEs) are used in many different applications, such as photovoltaics, optoelectronics, and display production, as well as in the automotive industry for windows with heaters and even in highly future-oriented applications of smart home construction, namely, smart windows.^1,2^ The main challenge is to adapt the manufacturing method to the required application and hence to find a balance between electrode transmission and conduction. These parameters will vary depending on the manufacturing method used for the TE.^3^

In general, we can distinguish two types of TEs. In short, they can be called planar and patterned. In the former, the whole surface is covered by a continuous conductive material, e.g., indium tin oxide (ITO),^4,5^ fluorine-doped tin oxide,^6^ graphene,^7^ a thin metal,^8^ a polymer layer,^9^ or other conductive films. The latter are transparent due to the very narrow width of the pattern, which can even be made of a nontransparent material; since the material only slightly covers the substrate surface, the entire electrode remains semitransparent.^10^ Those patterns can take the shape of squares, circles, polygons, or other regular or irregular intersecting lines. They must intersect to ensure good contact with each other and thus the good conductivity of the whole pattern, which creates the electrode.

Regular shapes such as mesh-shaped patterns are very common in the context of transparent electrodes and can be manufactured using many different methods, usually with tunable line width and spacing.^11−13^ Polygons, circles,^14,15^ and other regular shapes always require predesign to plan the movement of the printer head, laser head, or other device used in the method or, in the case of photolithography, to make the mask. Regular shapes have the advantage easily predicted TE mechanisms of action due to the simple design that can be simulated. However, a disadvantage that may occur is the Moiré phenomenon induced by the metal grids, which has been observed in some TE applications.^16^

Irregular shapes that create TE are not that common and usually require specific manufacturing methods such as the biomimic template reported by Jia et al.^17^ Additionally, irregularities are predominantly observed on the nanoscale, especially in methods utilizing nanowires. Silver nanowires are currently the most frequently reported nanomaterial.^18−20^

Meanwhile, achievements related to the production of TEs with irregular shapes on the macroscale are rarely reported. Such shapes require an unnecessary expenditure of energy and time to design a different pattern each time. It is challenging to obtain a shape with such a high degree of randomness, although such attempts have been made using the self-assembly process of Ag nanoparticles.^21,22^ Those TEs achieved very good transmission and resistivity parameters. However, it is difficult to create a homogeneous surface with this method because of differences in the layer morphology. Another idea for spontaneous shape formation is layer cracking. This technique was used by Rao et al. to obtain ITO-free organic solar cells,^23^ Cui et al. in the application of a thermochromic device,^24^ and Han et al. for the fabrication of a touch-screen device.^25^ These irregular shapes were also studied and subjected to simulations by Kim et al.^26^ A review summarizing the different types of transparent patterned conductors was also published by Gao et al.^27^ The present challenge for electrodes with irregular shapes is to move individual stages to production-scale and make them commercially feasible.

In this work, we present a method that allows the production of TEs with irregular, unique shapes on the macroscale while simultaneously requiring minimal effort to produce an electrode of this shape. We are focusing on entirely new applications of TEs, which we would like to emphasize were not previously published in this context.

## Results and Discussion

**Figure 1:**
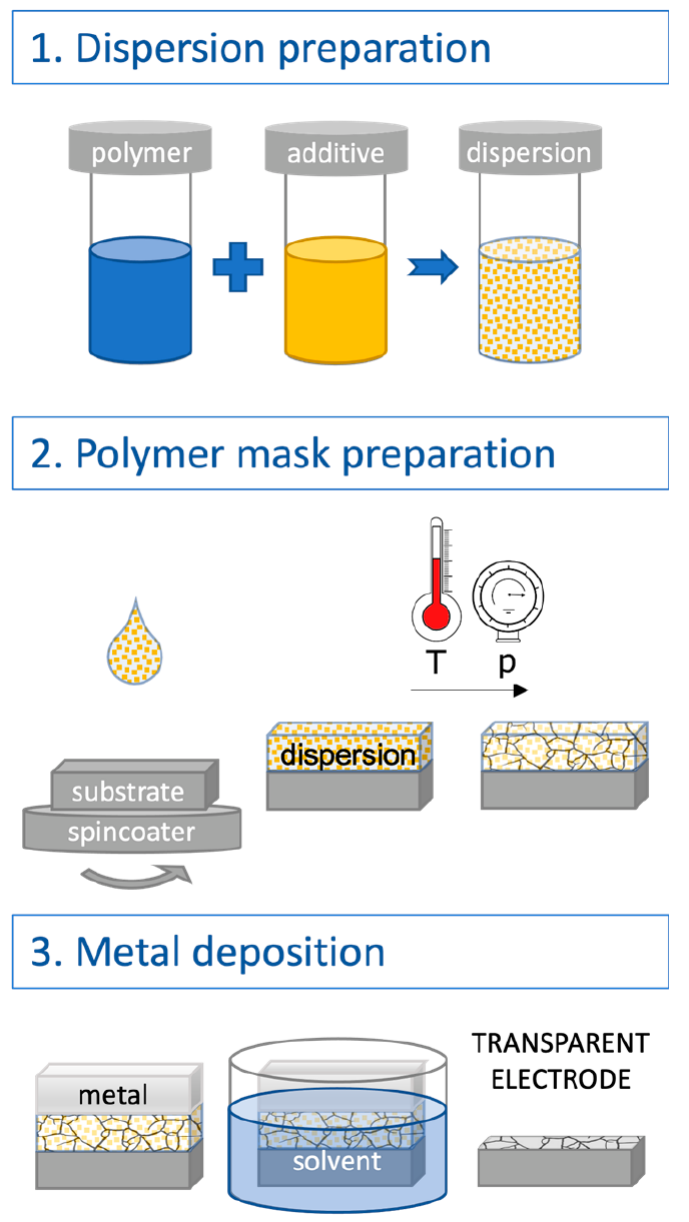
Scheme describing the procedure for manufacturing a transparent electrode using the cracking-induced technique.

Figure 1 shows a schematic diagram of the TE production procedure, starting with the preparation of the dispersion, continuing with the spin-coating and conditioning of the polymer mask, and ending with the deposition of the metal layer.

In the first step of the cracking-induced technique, which is called dispersion preparation in Figure 1, an appropriate polymer was chosen to constitute a matrix for a dispersion of nanoparticles that would later induce the cracking of that polymer. At the same time, this polymer should form a thin layer during spin-coating and should also be easily soluble for use as a disposable lift-off mask. All these requirements were met by the polyvinylpyrrolidone (PVP) powder with an average molecular weight of 10 000 that was purchased from Sigma-Aldrich. The concentration of PVP dissolved in ethanol was set at 0.5 g/mL. A vortex and a magnetic stirrer were used to help speed up the dissolution. As a nanodispersion, zinc oxide (ZnO) in ethanol was used because it was a readily available commercial reagent. It was bought from Sigma-Aldrich with 40 wt % nanoparticles and a particle size <130 nm. Due to the compatibility of the solvents of the two materials, it is easier to combine them because PVP is soluble in ethanol. To create a uniform polymer layer without agglomerates, this mixture was first homogenized for 6 min. This ensured a better dispersion of nanoparticles in the polymer and a longer suspension stability. The temperature during the homogenization process, measured with a thermometer immersed in the liquid, was 55 °C. The dispersion was stable for three days. After that time, the vortex was used to reunite the phases.

In the second step of the polymer mask preparation shown in Figure 1, 300 μL of the dispersion was spin-coated onto a transparent substrate at 4000 rpm for 40 s (POLOS). The layer thickness was measured on a Bruker Dektak contact profilometer. The sample was then placed onto hot plate and heated to 80 °C for 10 min to intentionally induce cracking. Higher temperatures up to 150 °C were also tested, but they did not caused any changes in the cracking process, proving that the evaporation temperature of the solvent (80 °C for ethanol) has a crucial impact. After that, the sample was placed under reduced pressure three times to intensify the shrinkage, which is a standard procedure to eliminate any moisture from a sample.

**Figure 2:**
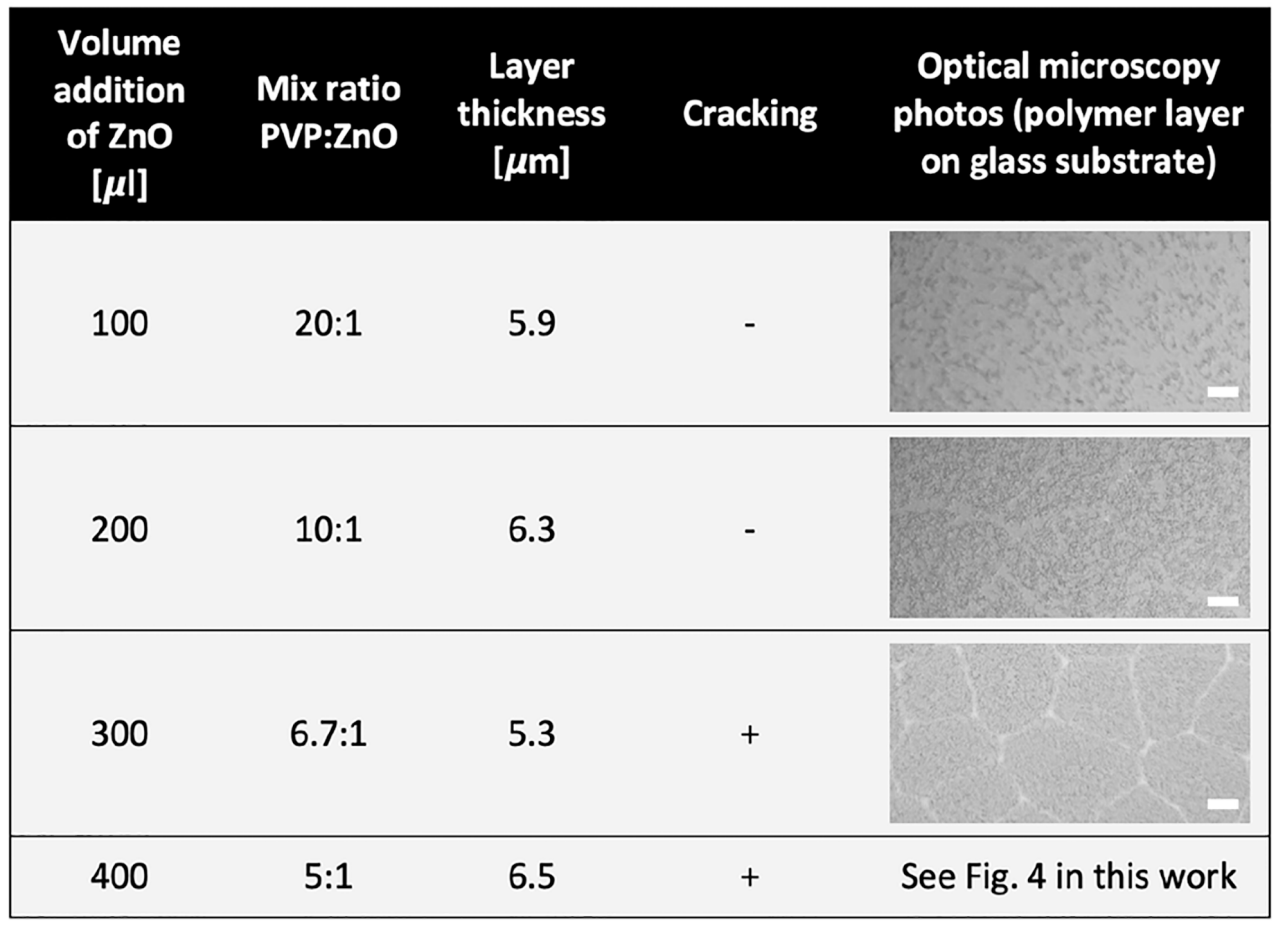
Results showing the effect of the amount of added ZnO on the induction of PVP polymer film cracking. The scale bar on the microscope pictures represents 20 μm.

To examine the cracking process, mix ratios of PVP to ZnO were studied. Keeping a constant amount of PVP polymer matrix equal to 2 mL, the amount of added ZnO was gradually increased by 100 μL. All layers were prepared according to the procedure described, and the results are summarized in Figure 2.

In case of the addition of 100 μL of the nanodispersion, no cracking was observed. For an addition of 200 μL, some domains had started to be created, but the layer remained continuous. The 300 μL addition resulted in cracking, but the spaces between the resulting domains were not yet visible. Finally, for the 400 μL addition, a satisfactory cracking effect was achieved because there were already visible spaces between the domains where the metallic material could enter during physical evaporation. Therefore, the PVP to ZnO ratio was set as 5:1.

In the final step (metal deposition in Figure 1), the prepared polymer mask was placed in a vacuum chamber to deposit the metallic layer in the MBraun evaporator. A 40 nm titanium adhesive layer was used under the 200 nm target silver layer. The resistive thermal evaporation system (for silver) and the electron beam (for titanium) were used with the following parameters: rate of 5 Å/s and pressure of 2 × 10^–6^ mbar. The metal was deposited all over the surface, including the spaces between the cracks. Since the PVP polymer is water-soluble, the substrate with the cracked polymer coating and the silver layer was immersed in water, which dissolved the PVP and caused the lift-off of the metallic layer. The remaining metallic part reproduced the shape of the cracks, creating a semitransparent electrode. The well-cleaned sample was then dried with nitrogen and subjected to optical and electrical analysis.

**Figure 3:**
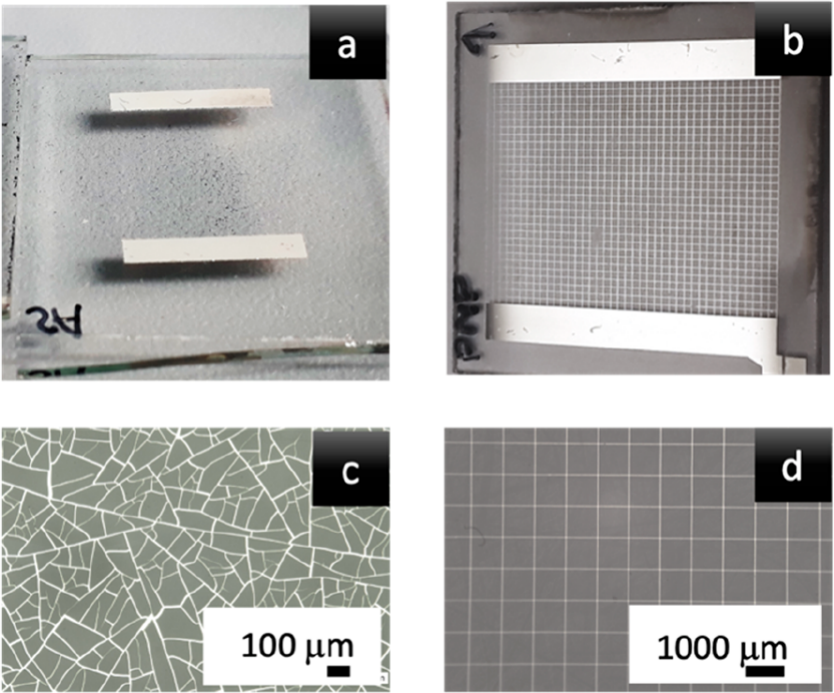
Comparison of (a) an electrode with a self-organized shape and (b) an electrode obtained by designing the mesh shape. (c and d) Photographs of the electrodes in panels a and b, respectively. Photographs were taken on an optical microscope.

The manufactured electrode is presented in Figure 3a and compared to the classical mesh-shaped semitransparent electrode^13^ (Figure 3b). The electrode obtained by inducing cracks in the polymer mask is characterized by unique shapes. Both types of electrodes are juxtaposed with each other in Figure 3 to show the shape differences.

**Figure 4:**
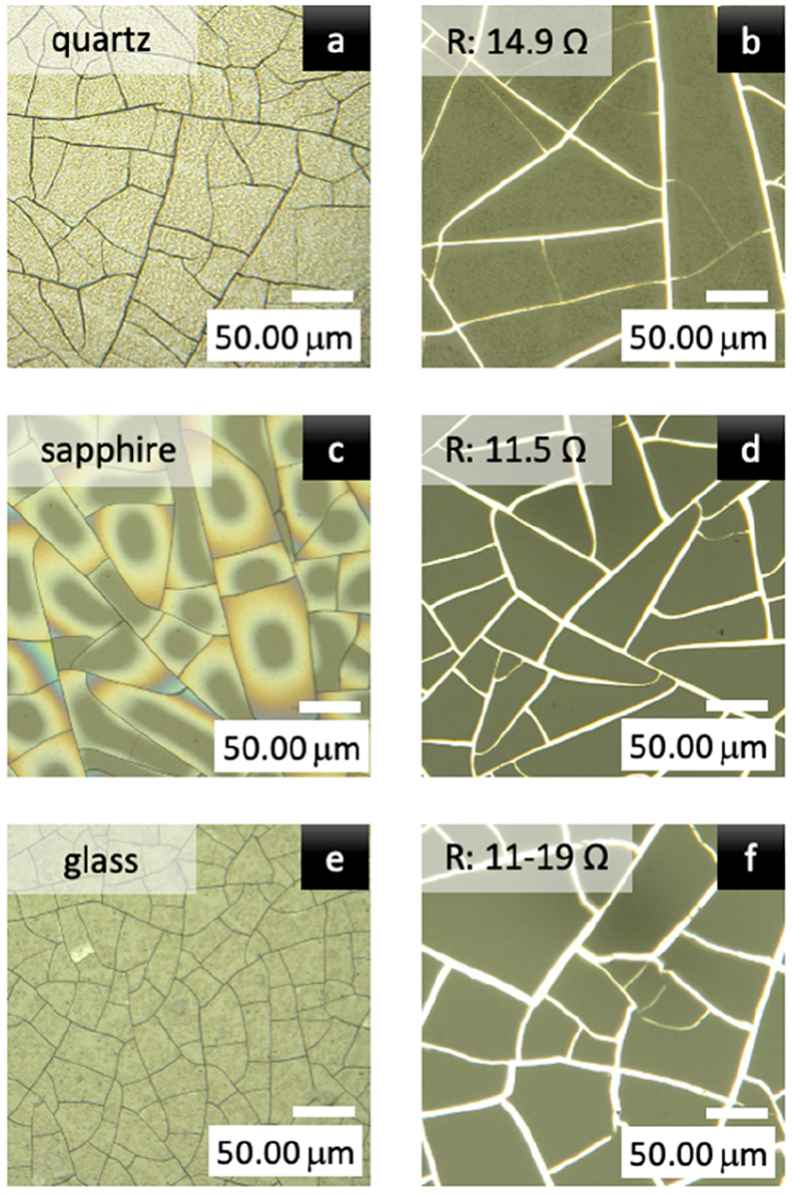
Optical microscope images of a polymer layer with the ZnO additive after heating at 80 °C on (a) quartz, (c) sapphire, and (e) glass substrates. Optical microscope images of the obtained semitransparent electrodes on the corresponding (b) quartz, (d) sapphire, and (f) glass substrates.

Different substrates were examined to check for the occurrence of the fracture process. Three transparent materials were tested: sapphire, glass, and quartz (see Figure 4).

For each of the tested substrates, crack-induced electrodes were able to be manufactured. To compare the obtained results, the key parameters of the TEs, namely, their transparency and conductivity (resistivity), were measured.

**Figure 5:**
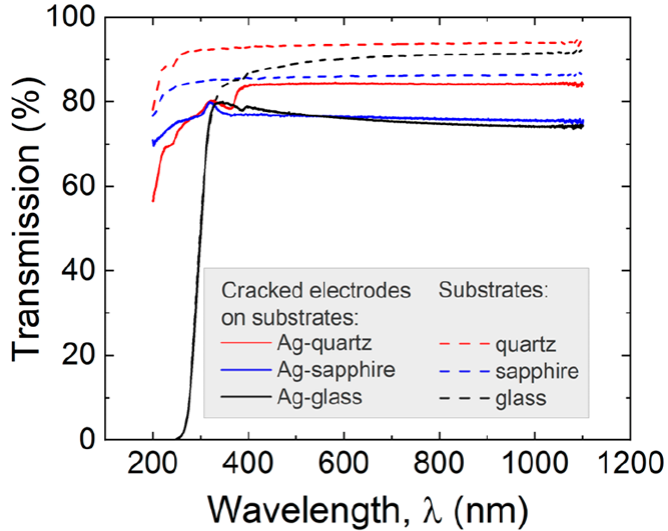
Transmission spectra of pure substrates (dashed lines) and substrates with deposited electrodes (solid lines). Differences in light transmission for quartz, sapphire, and glass are equal to 11.9%, 16.9%, and 11.1%, respectively. These differences can be attributed to the silver paths. For the measurement of the TE light transmission, a FILMETRICS F10-RT reflectometer was used.

In the case of transmission, pure substrates were examined first and then electrodes were made on these substrates. The initial transmission differs for each material. However, the changes in transmission before and after electrode insertion were almost the same, around 10–15% (Figure 5). The quartz from the Continental Trade Company performed the best in terms of optical and mechanical parameters while maintaining a reasonable price.

From these results, it can be seen that the spectral range of TE transmission can be regulated by the substrate material. Moreover, this range is wider for all materials in comparison to that of ITO, a popular material for transparent electrodes.^4^

Resistance (*R*), the second crucial parameter of a TE, was measured using the two-terminal method^28−30^ at a distance (*L*) of 11 mm. *R* ranged from 10 to 15 Ω, and these results were reproducible for various substrates. Using eq 1, the resistivity of the electrode (ρ) made on the glass substrate (with *R* = 15 Ω) was calculated to be 27 × 10^–7^ Ωm by considering the geometric sizes of the electrode (width *W* = 10 mm) and the average estimated pattern thickness (*t* = 200 nm).
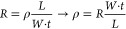
1

The sheet resistance *R*_S_ for this electrode, as defined by eq 2,^29^ is 13.65 Ω/sq.
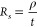
2

**Figure 6:**
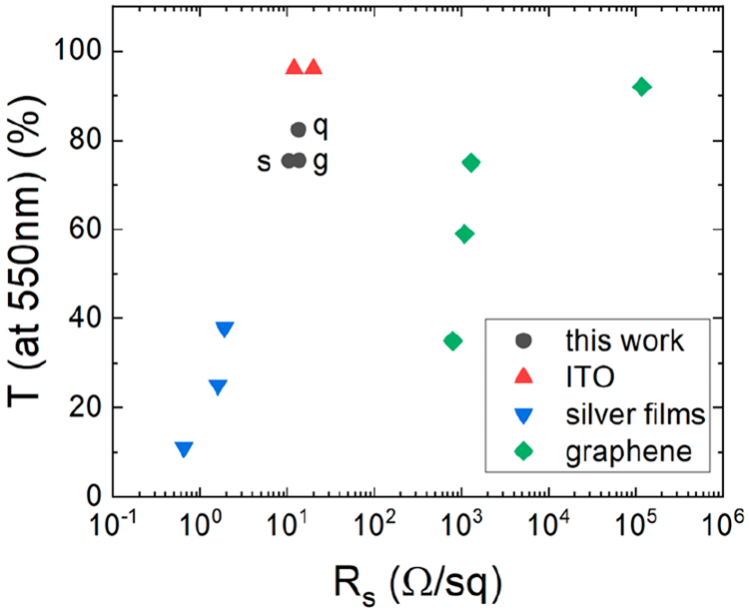
Transmittance (*T*) (550 nm) plotted as a function of the sheet resistance for graphene,^33^ commercial indium tin oxide (ITO),^34,35^ evaporated silver, and patterned silver described in this work (sapphire (s), quartz (q), and glass (g) substrates).

In the case of sapphire, *R*_S_ = 10.45 Ω/sq; in the case of quartz, *R*_S_ = 13.55 Ω/sq. These results are compared with the sheet resistances of other TEs in Figure 6.^31,32^

TEs with these unique, uneven patterns can be used as before in all TE applications, including contactless electroreflectance and surface photovoltage.^12^ However, these wire-like TEs also gain a new, additional applications due to their individual character in a new area where properties such as transparency, conductivity, and uniqueness are needed. An example is transparent electromagnetic interference filters, which were investigated by Shen et al. using random-pattern metal-mesh.^36^ Another interesting application of these TE could be prevention of the Moiré phenomenon so often present in the case of regular mesh-shaped TEs.^16^ This distinctiveness can be used independently or with accompanying high transmission in applications we do not even know yet.

**Figure 7:**
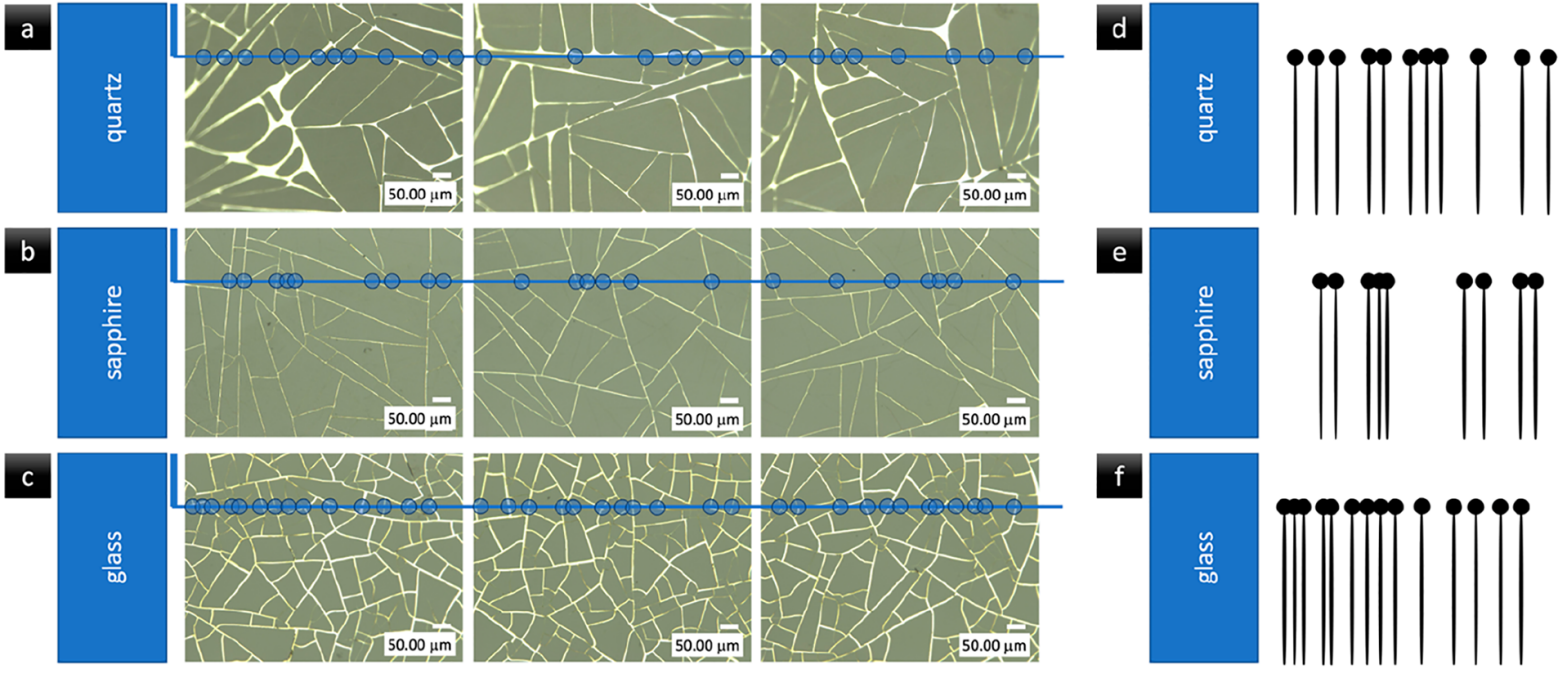
Optical images of transparent wire-like electrodes on the following substrates: (a) quartz, (b) sapphire, and (c) glass. (d–f) Illustration of the idea of coding the electrodes for each substrate; only the code for the first electrode of each material is presented.

TEs of unique shapes can be scanned and compared over the whole surface but also can be analyzed using the intersections with only one horizontal line on the initial comparative stage; like in case of fingerprint description, only some characteristic types of minutiae are considered. Those solutions for identifying a particular TE can find applications in areas such as coding, security, anticounterfeiting, plagiarism protection, marking, and readability. The idea of the encryption mechanism is illustrated in Figure 7.

As shown in Figure 7, a lower degree of compaction occurs for smoother substrate surfaces and a higher degree of compaction occurs for more rough, porous surfaces. Based on the coding properties of TEs, there are other new areas of possible applications such as logistics, where barcodes are used to identify particular products.

The undeniable advantage of using electrodes for encryption is their uniqueness, which gives confidence that the code will not be broken because the process itself occurs spontaneously. There are many possibilities for encryption thanks to the ability to control the size of the surface, which is limited only by the devices that apply the layers (spin-coater and vacuum chamber), using two properties of TEs: electrical and optical. An example of an encoding mechanism could be a method for determining where a horizontal line is cut off (in the example presented, the line is located one-third of the height from the top of the photo) and then determining the intersection points. The points of intersection can be determined by an optical method such as that presented in the figure but can also be read by an electrical probe, which upon contact with the path will conduct and record the position of the path. However, in the case of encryption using such TEs, the problem may be the difficulty of recovering lost data or the things that can be encrypted in this way. It should be pointed out that each possible application should be preceded by the optimization of the TE for that application, e.g., increasing the durability of the electrode by applying protective layers for methods that may cause mechanical damage, protecting against temperature extremes when using TEs in variable conditions, or matching the size of the substrate to the microscope holder or encoding station.

It is worth emphasizing that these irregular shapes can be obtained by lithographic techniques; however, these techniques would require the production of a mask with a different shape every time, which is uneconomic. In addition, printing techniques will not be suitable for reproducing such irregular shapes because the problem of overlapping paths on the intersections occurs, which leads to a heterogeneous surface over the entire electrode. In this case, our method is favorable, as it offers the possibility of producing such multifunctional electrodes quickly and cheaply thanks to its simple mechanism of producing a mask with a unique shape.

## Conclusions

In conclusion, we developed a novel technique for manufacturing TEs using standard materials. These electrodes are highly transparent (82.4%) in a broad spectral range, have transmission that can be regulated by the substrate selection, and possess low resistivity (27.3 × 10^–7^ Ωm). The method can be easily scaled up, since the structuring step is omitted. With these parameters, our TE is useful in well-known applications such as solar cells, touch panels, light-emitting diodes, transparent heaters, and others. Despite the fact that the electrode meets the requirements for existing applications because of its electrical and optical properties, it can also gain additional feature as a unique marker. We are fully convinced that TEs of irregular patterns can open up a completely new spectrum of application perspectives.
